# Supplementary figures and images for: Nitrogenase resurrection and the evolution of a singular enzymatic mechanism
Source: eLife. 2023 Feb 17;12:e85003. doi: 10.7554/eLife.85003 (PMC9977276; doi:10.7554/eLife.85003)

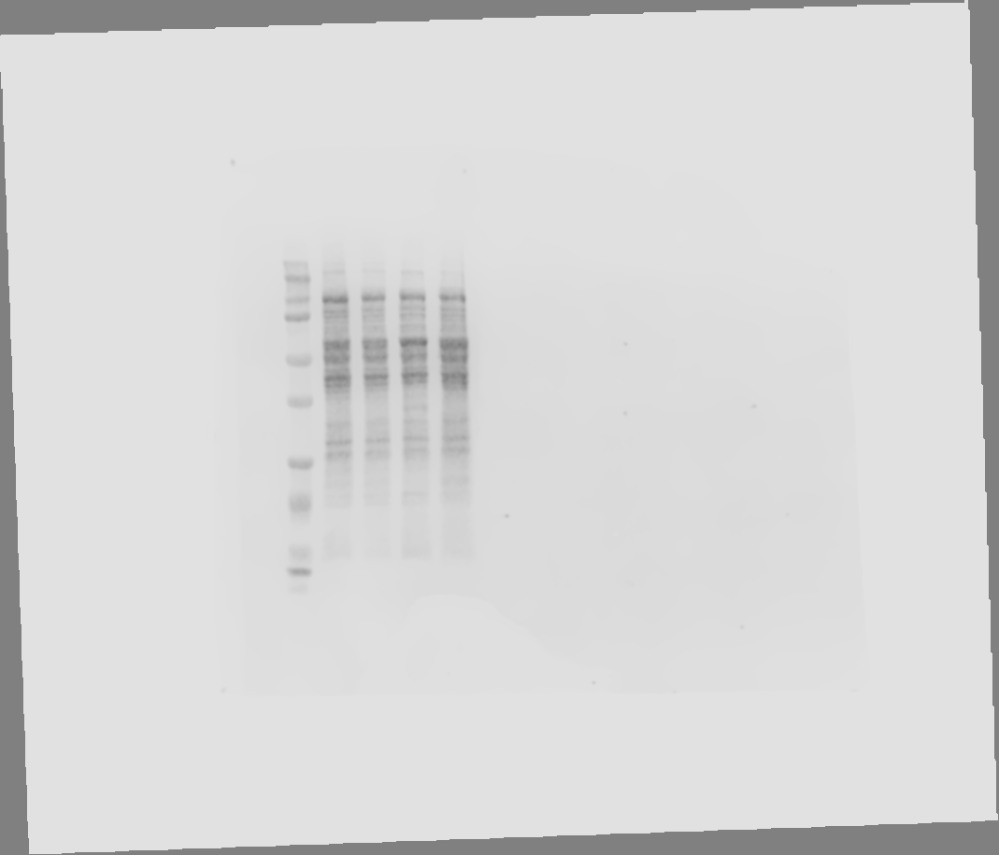

Supplement: Figure 3—source data 4. [file elife-85003-fig3-data4.zip › Figure3-SourceData4.tif]

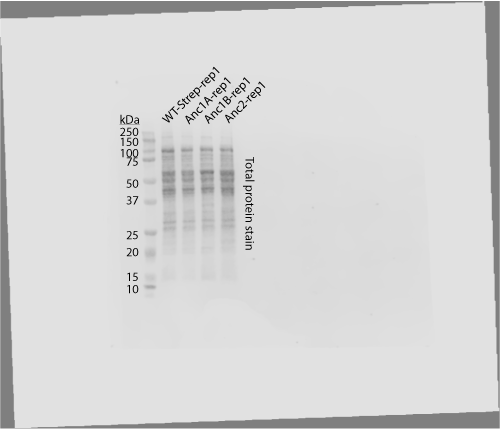

Supplement: Figure 3—source data 4. [file elife-85003-fig3-data4.zip › Figure3-SourceData4_labeled.tif]

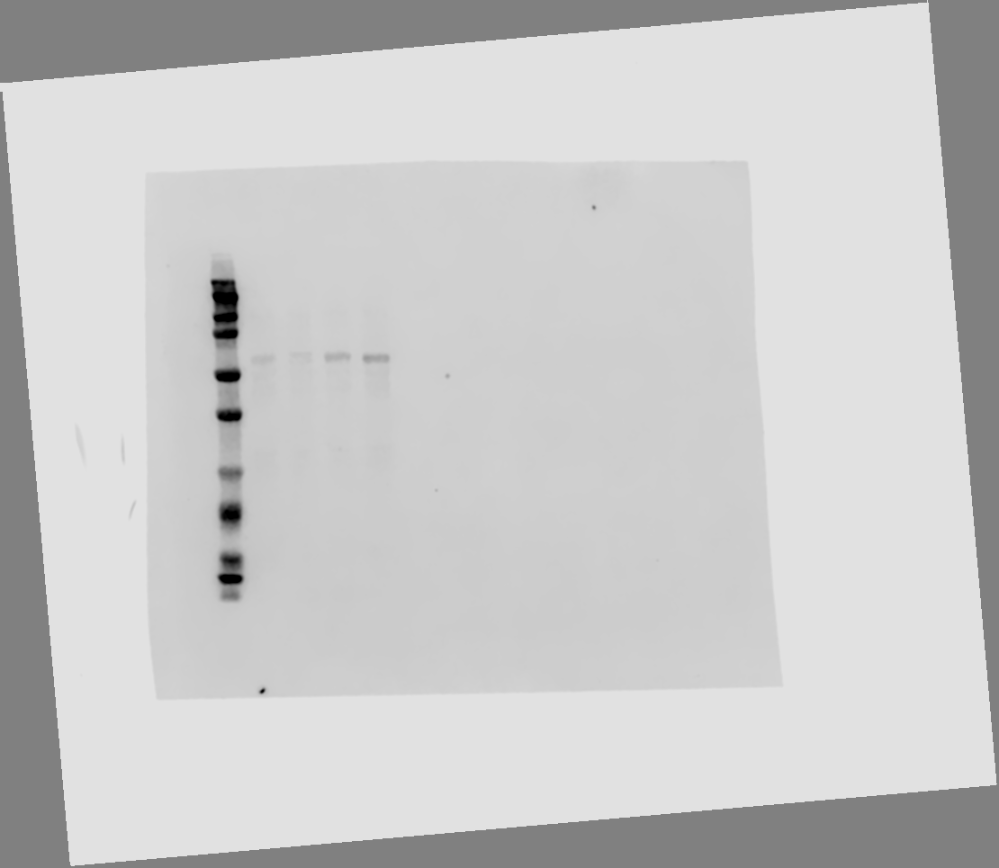

Supplement: Figure 3—source data 5. [file elife-85003-fig3-data5.zip › Figure3-SourceData5.tif]

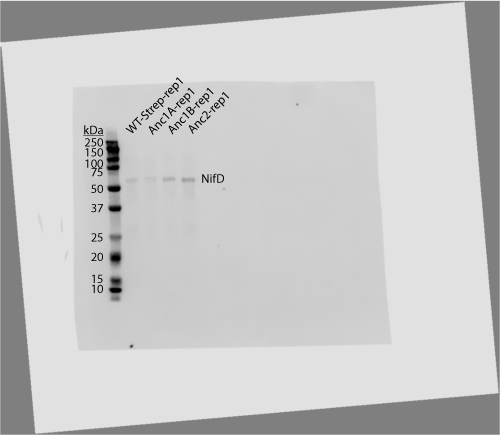

Supplement: Figure 3—source data 5. [file elife-85003-fig3-data5.zip › Figure3-SourceData5_labeled.tif]

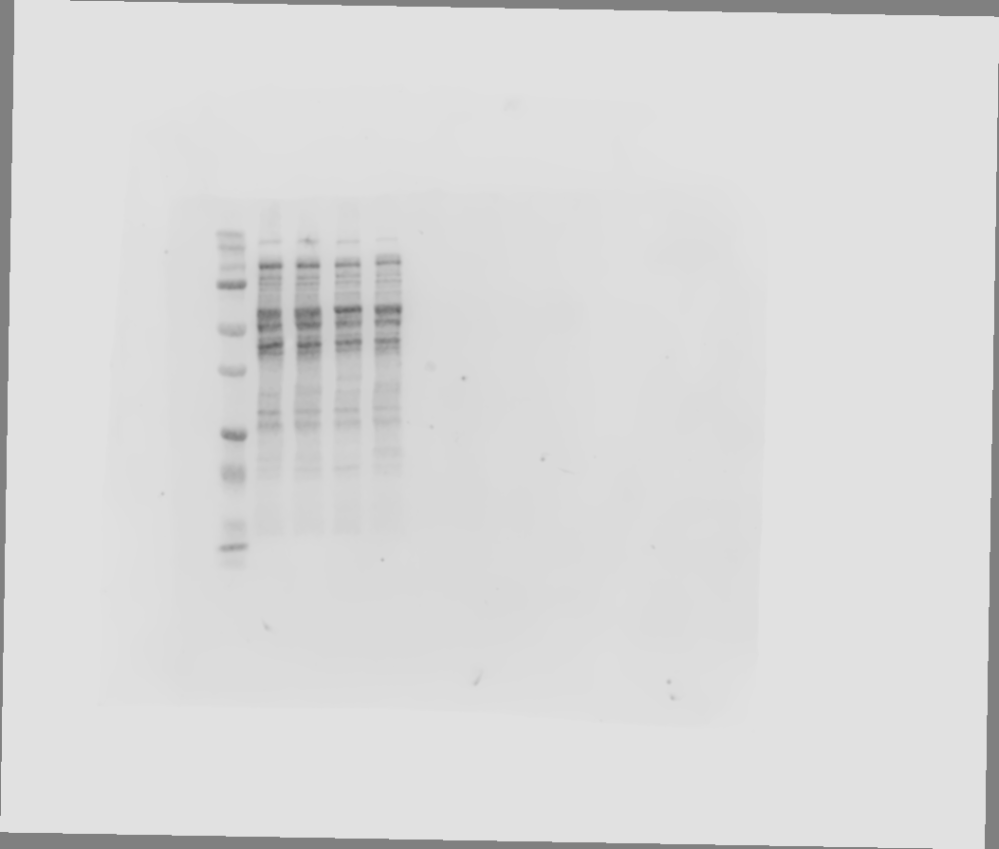

Supplement: Figure 3—source data 6. [file elife-85003-fig3-data6.zip › Figure3-SourceData6.tif]

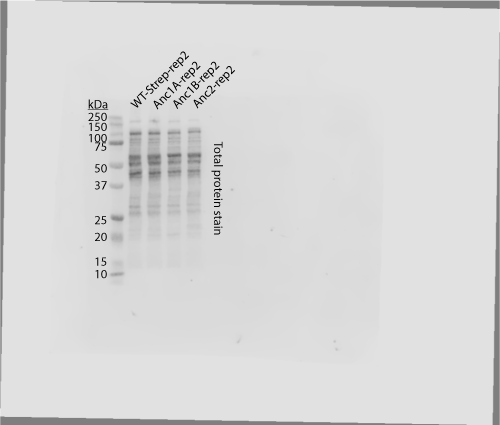

Supplement: Figure 3—source data 6. [file elife-85003-fig3-data6.zip › Figure3-SourceData6_labeled.tif]

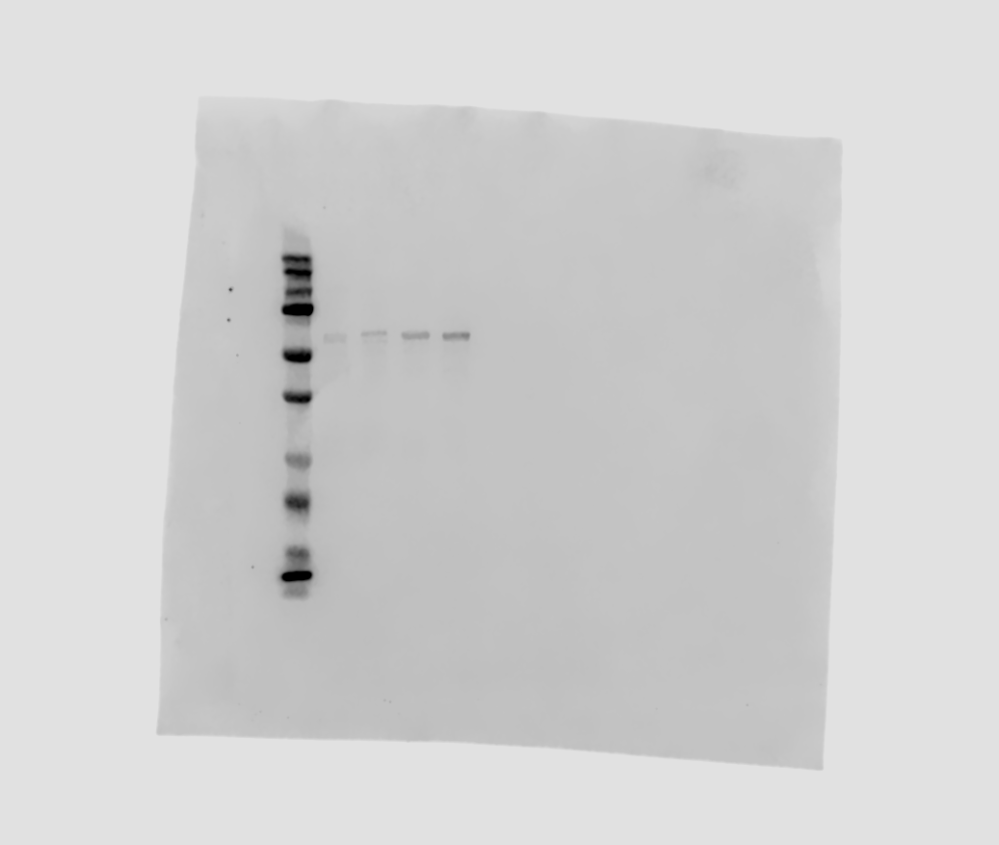

Supplement: Figure 3—source data 7. [file elife-85003-fig3-data7.zip › Figure3-SourceData7.tif]

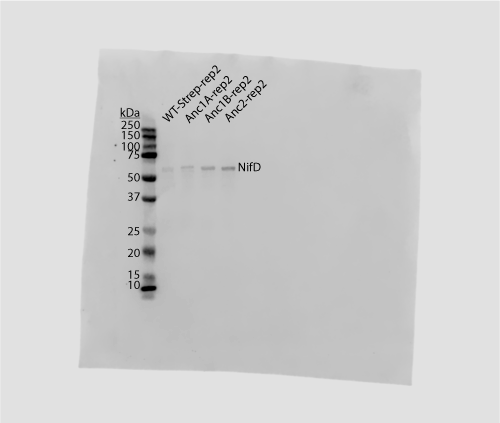

Supplement: Figure 3—source data 7. [file elife-85003-fig3-data7.zip › Figure3-SourceData7_labeled.tif]

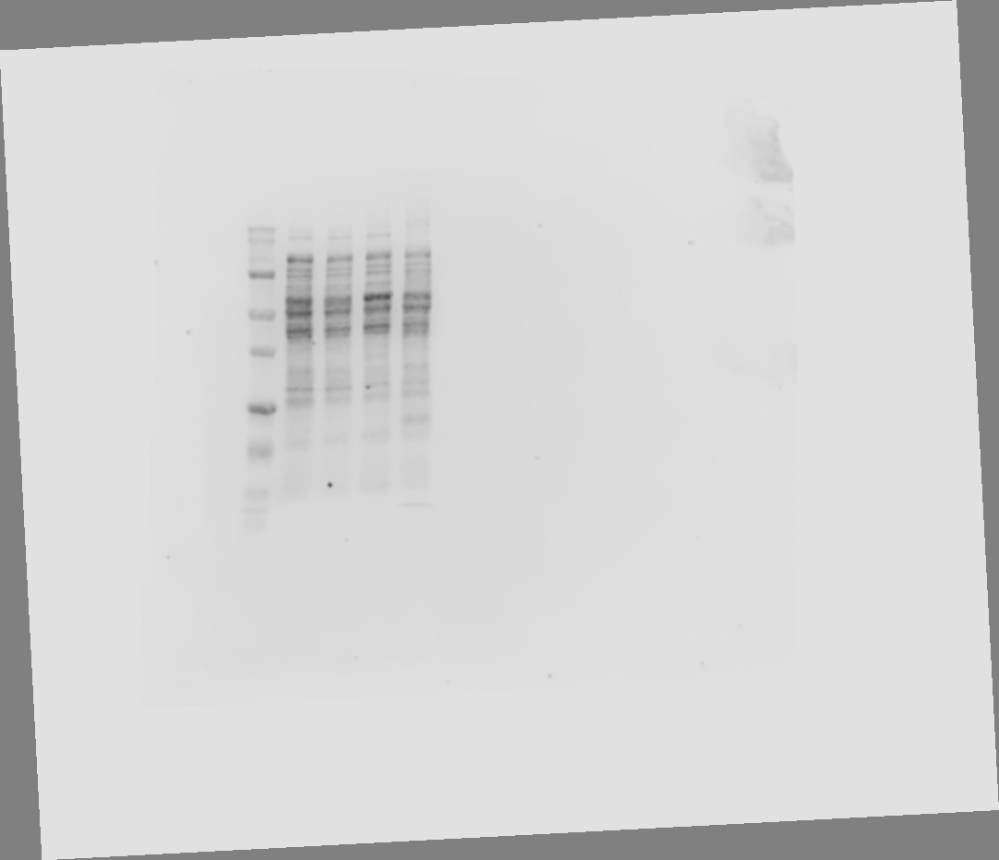

Supplement: Figure 3—source data 8. [file elife-85003-fig3-data8.zip › Figure3-SourceData8.tif]

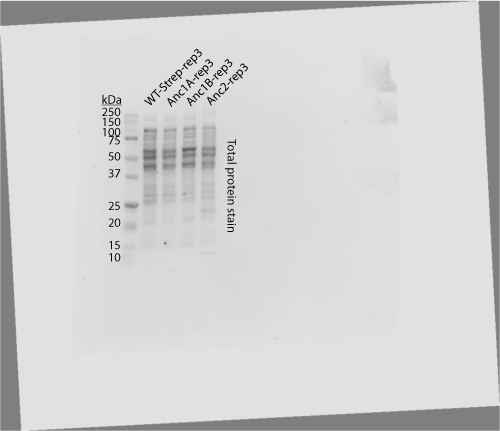

Supplement: Figure 3—source data 8. [file elife-85003-fig3-data8.zip › Figure3-SourceData8_labeled.tif]

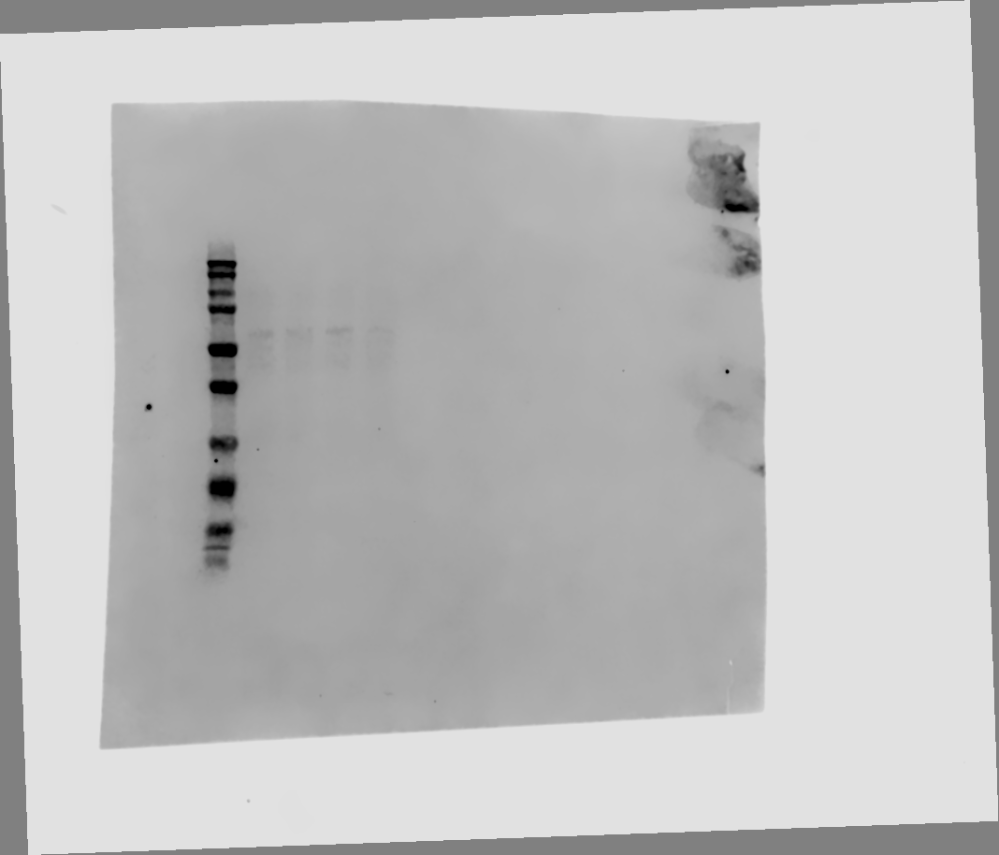

Supplement: Figure 3—source data 9. [file elife-85003-fig3-data9.zip › Figure3-SourceData9.tif]

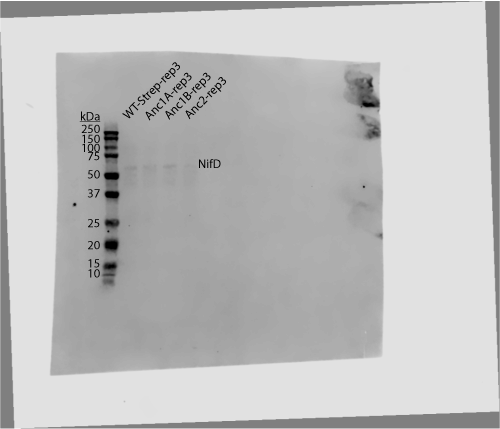

Supplement: Figure 3—source data 9. [file elife-85003-fig3-data9.zip › Figure3-SourceData9_labeled.tif]

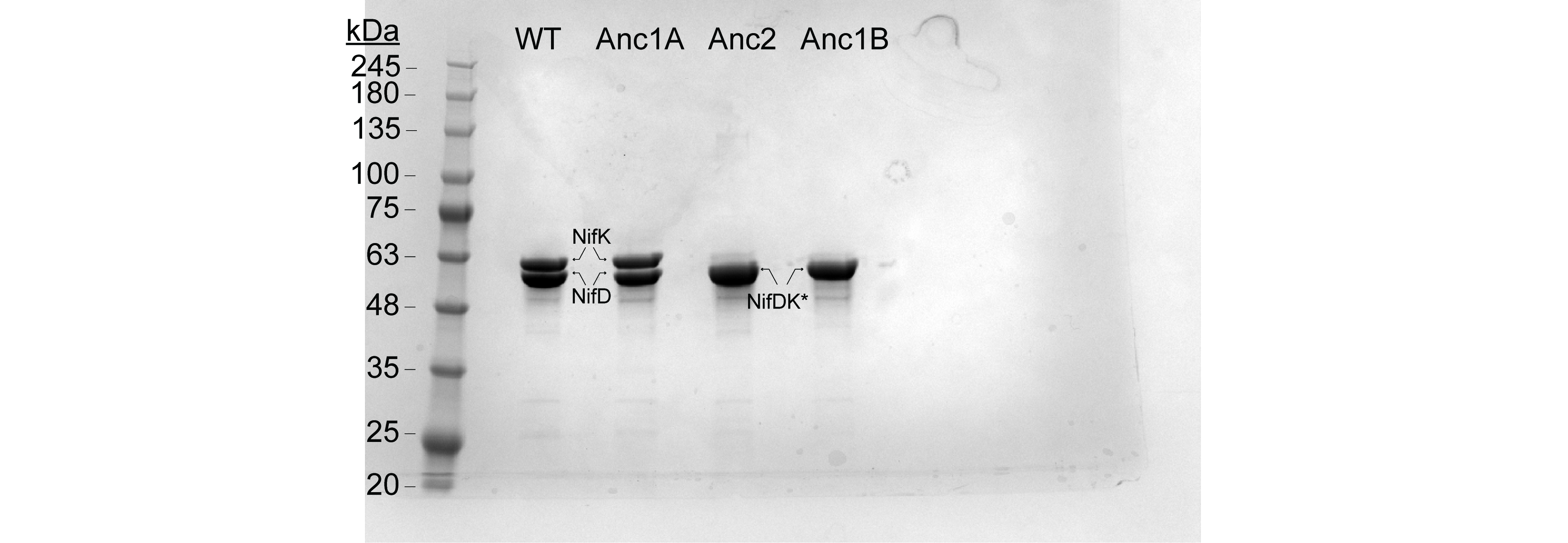

Supplement: Figure 4—figure supplement 1—source data 1. [file elife-85003-fig4-figsupp1-data1.zip › FigureS4-SourceData_labeled.tif]
