# Supplementary material for: Nitrogenase resurrection and the evolution of a singular enzymatic mechanism
Source: eLife. 2023 Feb 17;12:e85003. doi: 10.7554/eLife.85003 (PMC9977276; doi:10.7554/eLife.85003)
Supplement: Supplementary file 1. — (a) Sequence characteristics of ancestral nitrogenase subunits. (b) Host taxa of nitrogenase and outgroup dark-operative protochlorophyllide oxidoreductase homologs included for phylogenetic analysis. (c) Strains and plasmids used in this study. (d) Primers used in this study. [file elife-85003-supp1.docx]

**SUPPLEMENTARY FILE 1**

**Supplementary File 1a. Sequence characteristics of ancestral nitrogenase subunits.**

| Subunit | % protein identity to WT | Mean site posterior probability | % sites with plausible alternate ancestral states | Substitutions at relatively conserved sites^†^ |
| --- | --- | --- | --- | --- |
| NifH^Anc1B^ | 94% | 0.99 | 1% | G181A |
| NifD^Anc1A,Anc1B^ | 89% | 0.97 | 3% | I47T, L56V**, C249V**, I355V*, F429Y** |
| NifK^Anc1B^ | 89% | 0.98 | 2% | R108K**, H396N** |
| NifD^Anc2^ | 83% | 0.95 | 2% | I47T, L56V**, C249V**, V273L, T305I, C324A, I355V*, M394L, F429Y**, F453Y |

*Active-site

**NifD:NifK interface

^†^Defined by a Consurf score > 7; see Materials and Methods

**Supplementary File 1b. Host taxa of nitrogenase and outgroup dark-operative protochlorophyllide oxidoreductase homologs included for phylogenetic analysis.**

| **Type** | **Organism** |
| --- | --- |
| Nif | Acetobacter peroxydans |
| Nif | Acetobacterium woodii |
| Nif | Acidiferrobacter sp. SPIII_3 |
| Nif | Acidihalobacter prosperus |
| Nif | Acidithiobacillus ferridurans |
| Nif | Actibacterium ureilyticum |
| Nif | Actinobacteria bacterium HGW-Actinobacteria-2 |
| Nif | Actinobacteria bacterium HGW-Actinobacteria-10 |
| Nif | Acuticoccus sp. PTG4-2 |
| Nif | Afifella pfennigii |
| Nif | Agaribacterium haliotis |
| Nif | Aliagarivorans marinus |
| Nif | Alkalibacter saccharofermentans |
| Nif | Alkaliflexus imshenetskii |
| Nif | Alkaliphilus metalliredigens |
| Nif | Alphaproteobacteria bacterium |
| Nif | Alteromonadaceae bacterium Bs31 |
| Nif | Alteromonadales bacterium BS08 |
| Nif | Ammonifex sp. |
| Nif | Amphritea atlantica |
| Nif | Anaeroarcus burkinensis |
| Nif | Anaerobacillus alkalidiazotrophicus |
| Nif | Anaerobacterium chartisolvens |
| Nif | Anaerocolumna jejuensis |
| Nif | Anaeromyxobacter sp. Fw109-5 |
| Nif | Anaerospora hongkongensis |
| Nif | Anaerosporobacter mobilis |
| Nif | Anaerosporomusa subterranea |
| Nif | Anaerotignum neopropionicum |
| Nif | Aneurinibacillus terranovensis |
| Nif | ANME-2 cluster archaeon |
| Nif | archaeon BMS3Abin16 |
| Nif | archaeon BMS3Bbin15 |
| Nif | Arcticibacter svalbardensis |
| Nif | Azoarcus sp. CC-YHH838 |
| Nif | Azotobacter vinelandii |
| Nif | Bacillus caseinilyticus |
| Nif | bacterium BMS3Abin10 |
| Nif | bacterium BMS3Abin12 |
| Nif | bacterium BMS3Abin13 |
| Nif | bacterium BMS3Bbin07 |
| Nif | Bacteroidales bacterium |
| Nif | Bacteroidales bacterium Barb6XT |
| Nif | Bacteroides graminisolvens |
| Nif | Bacteroides luti |
| Nif | Beggiatoa alba |
| Nif | Betaproteobacteria bacterium HGW-Betaproteobacteria-11 |
| Nif | Biostraticola sp. BGMRC 2031 |
| Nif | Blastochloris sp. GI |
| Nif | Blastopirellula marina |
| Nif | Bradyrhizobium oligotrophicum |
| Nif | Caldicellulosiruptor saccharolyticus |
| Nif | Calditerrivibrio nitroreducens |
| Nif | Candidatus Accumulibacter aalborgensis |
| Nif | Candidatus Achromatium palustre |
| Nif | Candidatus Acididesulfobacter diazotrophicus |
| Nif | Candidatus Acidulodesulfobacterium acidiphilum |
| Nif | Candidatus Argoarchaeum ethanivorans |
| Nif | Candidatus Azobacteroides pseudotrichonymphae |
| Nif | Candidatus Competibacteraceae bacterium |
| Nif | Candidatus Dadabacteria bacterium |
| Nif | Candidatus Electronema sp. GS |
| Nif | Candidatus Lambdaproteobacteria bacterium RIFOXYC1_FULL_56_13 |
| Nif | Candidatus Lambdaproteobacteria bacterium RIFOXYD2_FULL_50_16 |
| Nif | Candidatus Magnetoovum chiemensis |
| Nif | Candidatus Margulisbacteria bacterium GWD2_39_127 |
| Nif | Candidatus Marispirochaeta associata |
| Nif | Candidatus Methanolliviera hydrocarbonicum |
| Nif | Candidatus Methanolliviera sp. GoM_asphalt |
| Nif | Candidatus Methanoperedens nitroreducens |
| Nif | Candidatus Methanoperedens sp. BLZ2 |
| Nif | Candidatus Thiosymbion oneisti |
| Nif | Candidatus Viridilinea mediisalina |
| Nif | Carboxydocella sp. ULO1 |
| Nif | Celerinatantimonas diazotrophica |
| Nif | Cellulosilyticum lentocellum |
| Nif | Chlorobium chlorochromatii |
| Nif | Chloroflexales bacterium ZM16-3 |
| Nif | Chloroflexi bacterium |
| Nif | Chloroherpeton thalassium |
| Nif | Chromatiaceae bacterium 2141T.STBD.0c.01a |
| Nif | Chrysiogenes arsenatis |
| Nif | Clostridiales bacterium 43-6 |
| Nif | Clostridiales bacterium DRI-13 |
| Nif | [Clostridium] algidixylanolyticum |
| Nif | [Clostridium] fimetarium |
| Nif | Clostridium pasteurianum |
| Nif | [Clostridium] populeti |
| Nif | Cohaesibacter haloalkalitolerans |
| Nif | Coleofasciculus chthonoplastes |
| Nif | Coraliomargarita akajimensis |
| Nif | Cupriavidus sp. amp6 |
| Nif | cyanobacterium endosymbiont of Epithemia turgida |
| Nif | Cytophaga xylanolytica |
| Nif | Defluviimonas alba |
| Nif | Defluviitalea phaphyphila |
| Nif | Dehalobacter |
| Nif | Dehalococcoides mccartyi |
| Nif | Dehalogenimonas sp. WBC-2 |
| Nif | delta proteobacterium NaphS2 |
| Nif | Deltaproteobacteria bacterium |
| Nif | Deltaproteobacteria bacterium HGW-Deltaproteobacteria-4 |
| Nif | Deltaproteobacteria bacterium HGW-Deltaproteobacteria-8 |
| Nif | Dendrosporobacter quercicolus |
| Nif | Derxia gummosa |
| Nif | Desertifilum sp. IPPAS B-1220 |
| Nif | Desulfacinum infernum |
| Nif | Desulfallas arcticus |
| Nif | Desulfamplus magnetovallimortis |
| Nif | Desulfatibacillum aliphaticivorans |
| Nif | Desulfatitalea sp. BRH_c12 |
| Nif | Desulfobacca acetoxidans |
| Nif | Desulfobacteraceae bacterium |
| Nif | Desulfobacteraceae bacterium Eth-SRB1 |
| Nif | Desulfobacteraceae bacterium Eth-SRB2 |
| Nif | Desulfobacterales bacterium C00003106 |
| Nif | Desulfobacterium autotrophicum |
| Nif | Desulfobotulus alkaliphilus |
| Nif | Desulfobulbaceae bacterium |
| Nif | Desulfobulbaceae bacterium A2 |
| Nif | Desulfobulbaceae bacterium DB1 |
| Nif | Desulfobulbus elongatus |
| Nif | Desulfocarbo indianensis |
| Nif | Desulfocucumis palustris |
| Nif | Desulfocurvibacter africanus |
| Nif | Desulfofarcimen acetoxidans |
| Nif | Desulfofustis glycolicus |
| Nif | Desulfoglaeba alkanexedens |
| Nif | Desulfohalovibrio alkalitolerans |
| Nif | Desulfoluna spongiiphila |
| Nif | Desulfomicrobium apsheronum |
| Nif | Desulfomonile tiedjei |
| Nif | Desulfonatronospira thiodismutans |
| Nif | Desulfonatronovibrio hydrogenovorans |
| Nif | Desulfonatronum lacustre |
| Nif | Desulfopila aestuarii |
| Nif | Desulforegula conservatrix |
| Nif | Desulforhopalus singaporensis |
| Nif | Desulfosarcina cetonica |
| Nif | Desulfospira joergensenii |
| Nif | Desulfosporosinus fructosivorans |
| Nif | Desulfotalea sp. |
| Nif | Desulfotomaculum ferrireducens |
| Nif | Desulfovibrio desulfuricans |
| Nif | Desulfuribacillus alkaliarsenatis |
| Nif | Desulfurispora thermophila |
| Nif | Desulfurobacterium atlanticum |
| Nif | Desulfuromonadales bacterium GWC2_61_20 |
| Nif | Desulfuromonas acetexigens |
| Nif | Dethiobacter alkaliphilus |
| Nif | Dethiosulfatibacter aminovorans |
| Nif | Dissulfuribacter thermophilus |
| Nif | Draconibacterium sediminis |
| Nif | Dysgonomonas capnocytophagoides |
| Nif | Ectothiorhodospira haloalkaliphila |
| Nif | Ectothiorhodospiraceae bacterium |
| Nif | Elusimicrobia bacterium RIFOXYA2_FULL_50_26 |
| Nif | Ethanoligenens harbinense |
| Nif | Euhalothece natronophila |
| Nif | Fibrobacteres bacterium |
| Nif | filamentous cyanobacterium ESFC-1 |
| Nif | Firmicutes bacterium HGW-Firmicutes-1 |
| Nif | Firmicutes bacterium HGW-Firmicutes-8 |
| Nif | Fischerella sp. PCC 9605 |
| Nif | Fontibacillus panacisegetis |
| Nif | Frankia canadensis |
| Nif | Gaiellales bacterium |
| Nif | Gallionellales bacterium GWA2_60_18 |
| Nif | Gammaproteobacteria bacterium 28-57-27 |
| Nif | Geitlerinema sp. PCC 9228 |
| Nif | Geminicoccaceae bacterium |
| Nif | Geminisphaera colitermitum |
| Nif | Geoalkalibacter ferrihydriticus |
| Nif | Geobacter thiogenes |
| Nif | Geosporobacter ferrireducens |
| Nif | Geothermobacter sp. EPR-M |
| Nif | Geovibrio sp. L21-Ace-BES |
| Nif | Gloeocapsa sp. DLM2.Bin57 |
| Nif | Gordonibacter sp. 28C |
| Nif | Gorillibacterium timonense |
| Nif | Gracilibacter sp. BRH_c7a |
| Nif | Halanaerobium salsuginis |
| Nif | Haliea sp. SAOS-164 |
| Nif | Halocella sp. SP3-1 |
| Nif | Halorhodospira halochloris |
| Nif | Hartmannibacter diazotrophicus |
| Nif | Heliobacterium modesticaldum |
| Nif | Herbaspirillum frisingense |
| Nif | Holophaga foetida |
| Nif | Hungateiclostridium cellulolyticum |
| Nif | Hydrogenispora ethanolica |
| Nif | Hydrogenophilales bacterium 16-64-40 |
| Nif | Hydrogenophilales bacterium 28-61-23 |
| Nif | Ilyobacter polytropus |
| Nif | Kamptonema |
| Nif | Kiritimatiellales bacterium |
| Nif | Klebsiella pneumoniae |
| Nif | Labilibacter aurantiacus |
| Nif | Lachnospiraceae bacterium |
| Nif | Lachnospiraceae bacterium Marseille-P3773 |
| Nif | Lebetimonas sp. JS032 |
| Nif | Lentisphaerae bacterium GWF2_50_93 |
| Nif | Lentisphaerae bacterium GWF2_57_35 |
| Nif | [Leptolyngbya] sp. JSC-1 |
| Nif | Leptospirillum ferriphilum |
| Nif | Lucifera butyrica |
| Nif | Lutibacter agarilyticus |
| Nif | Lyngbya sp. PCC 8106 |
| Nif | magneto-ovoid bacterium MO-1 |
| Nif | Magnetovibrio blakemorei |
| Nif | Malonomonas rubra |
| Nif | Mangrovibacter sp. MFB070 |
| Nif | Mangrovibacterium diazotrophicum |
| Nif | Marinilabilia sp. WTE |
| Nif | Marinobacter sp. ES-1 |
| Nif | Mariprofundus ferrooxydans |
| Nif | Martelella sp. BGMRC2036 |
| Nif | Massilibacillus massiliensis |
| Nif | Megasphaera cerevisiae |
| Nif | Methanobacteriales archaeon HGW-Methanobacteriales-1 |
| Nif | Methanobacterium paludis |
| Nif | Methanobrevibacter curvatus |
| Nif | Methanocaldococcus infernus |
| Nif | Methanocella arvoryzae |
| Nif | Methanococcus maripaludis |
| Nif | Methanoculleus taiwanensis |
| Nif | Methanolacinia paynteri |
| Nif | Methanolobus psychrotolerans |
| Nif | Methanomassiliicoccus luminyensis |
| Nif | Methanomicrobiales archaeon HGW-Methanomicrobiales-1 |
| Nif | Methanophagales archaeon |
| Nif | Methanoregula boonei |
| Nif | Methanosarcina acetivorans C2A |
| Nif | Methanosphaerula palustris |
| Nif | Methanospirillum lacunae |
| Nif | Methanothermobacter thermautotrophicus |
| Nif | Methanothermococcus okinawensis |
| Nif | Methanothrix soehngenii |
| Nif | Methanotorris igneus |
| Nif | Methyloferula stellata |
| Nif | Methyloglobulus morosus |
| Nif | Methylomonas koyamae |
| Nif | Methyloprofundus sedimenti |
| Nif | Moorella thermoacetica |
| Nif | Natranaerovirga hydrolytica |
| Nif | Natronoflexus pectinivorans |
| Nif | Neorhizobium galegae |
| Nif | Nitrospirae bacterium |
| Nif | Nitrospirae bacterium GWC2_57_13 |
| Nif | Oceanospirillaceae bacterium |
| Nif | Opitutaceae bacterium EW11 |
| Nif | Orenia marismortui |
| Nif | Orenia metallireducens |
| Nif | Oscillochloris trichoides |
| Nif | Oxalobacter sp. |
| Nif | Oxobacter pfennigii |
| Nif | Paludibacter propionicigenes |
| Nif | Pantoea cypripedii |
| Nif | Parasporobacterium paucivorans |
| Nif | Pectinatus cerevisiiphilus |
| Nif | Pelobacter acetylenicus |
| Nif | Pelomonas saccharophila |
| Nif | Pelotomaculum sp. FP |
| Nif | Peptococcaceae bacterium BICA1-7 |
| Nif | Peptococcaceae bacterium BRH_c4a |
| Nif | Peptococcaceae bacterium CEB3 |
| Nif | Peptococcaceae bacterium SCADC1_2_3 |
| Nif | Petrocella atlantisensis |
| Nif | Petroclostridium xylanilyticum |
| Nif | Phaeospirillum fulvum |
| Nif | Phormidium sp. HE10JO |
| Nif | Phycisphaeraceae bacterium |
| Nif | Planctomycetaceae bacterium |
| Nif | Planctomycetes bacterium |
| Nif | Planctomycetes bacterium Q31b |
| Nif | Planctomycetes bacterium V7 |
| Nif | Planktothrix tepida |
| Nif | Pleomorphomonas carboxyditropha |
| Nif | Polaromonas naphthalenivorans |
| Nif | Prevotella oryzae |
| Nif | Propionibacterium cyclohexanicum |
| Nif | Propionispira arboris |
| Nif | Propionivibrio dicarboxylicus |
| Nif | Prosthecochloris sp. GSB1 |
| Nif | Proteobacteria bacterium CG1_02_64_396 |
| Nif | Pseudobacteroides cellulosolvens |
| Nif | Pseudoclostridium thermosuccinogenes |
| Nif | Pseudodesulfovibrio hydrargyri |
| Nif | Rhizobiales bacterium |
| Nif | Rhodobacteraceae bacterium |
| Nif | Rhodoblastus acidophilus |
| Nif | Rhodocyclaceae bacterium |
| Nif | Rhodopila globiformis |
| Nif | Rhodopseudomonas palustris |
| Nif | Rhodospirillaceae bacterium BRH_c57 |
| Nif | Rhodothalassium salexigens |
| Nif | Rhodovibrio salinarum |
| Nif | Robinsoniella sp. MCWD5 |
| Nif | Roseiflexus castenholzii |
| Nif | Roseofilum reptotaenium AO1-A |
| Nif | Roseospirillum parvum |
| Nif | Ruminiclostridium hungatei |
| Nif | Sediminispirochaeta smaragdinae |
| Nif | Seleniivibrio woodruffii |
| Nif | Serpentinicella alkaliphila |
| Nif | Serratia sp. ATCC 39006 |
| Nif | Skermanella aerolata |
| Nif | Smithella sp. SC_K08D17 |
| Nif | Sodalis sp. 159R |
| Nif | Solimonas aquatica |
| Nif | Spirochaeta cellobiosiphila |
| Nif | Spirochaeta thermophila |
| Nif | Spirochaetaceae bacterium |
| Nif | Spirochaetes bacterium GWB1_27_13 |
| Nif | Spirochaetes bacterium GWB1_36_13 |
| Nif | Spirochaetes bacterium GWE1_32_154 |
| Nif | Sporobacter termitidis |
| Nif | Sporolactobacillus terrae |
| Nif | Sporomusa termitida |
| Nif | Sulfuricurvum kujiense |
| Nif | Sulfurimonas sp. RIFOXYD12_FULL_33_39 |
| Nif | Sulfurivermis fontis |
| Nif | Sulfurovum sp. FS08-3 |
| Nif | Syntrophobacter fumaroxidans |
| Nif | Syntrophomonas zehnderi |
| Nif | Syntrophorhabdaceae bacterium PtaU1.Bin034 |
| Nif | Syntrophothermus lipocalidus |
| Nif | Syntrophus gentianae |
| Nif | Telmatospirillum siberiense |
| Nif | Terrimicrobium sacchariphilum |
| Nif | Thermacetogenium phaeum |
| Nif | Thermicanus aegyptius |
| Nif | Thermoanaerobacterium thermosaccharolyticum |
| Nif | Thermodesulfitimonas autotrophica |
| Nif | Thermodesulforhabdus norvegica |
| Nif | Thermodesulfovibrio aggregans |
| Nif | Thioflavicoccus mobilis |
| Nif | Thioploca ingrica |
| Nif | Thiorhodococcus drewsii |
| Nif | Tolumonas lignilytica |
| Nif | Treponema primitia |
| Nif | uncultured archaeon |
| Nif | Varunaivibrio sulfuroxidans |
| Nif | Verrucomicrobia bacterium S94 |
| Nif | Verrucomicrobia bacterium Tous-C9LFEB |
| Nif | Verrucomicrobiae bacterium DG1235 |
| Nif | Vibrio aerogenes |
| Nif | Vulcanococcus limneticus |
| Nif | Wolinella succinogenes |
| Nif | Youngiibacter fragilis |
| Nif | Zymomonas mobilis |
| Vnf | Archaeoglobus sp. |
| Vnf | Azomonas agilis |
| Vnf | Azotobacter vinelandii |
| Vnf | Clostridium pasteurianum |
| Vnf | Desulfobacter curvatus |
| Vnf | Ethanoligenens harbinense |
| Vnf | Lucifera butyrica |
| Vnf | Methanosarcina acetivorans C2A |
| Vnf | Methylocystaceae bacterium |
| Vnf | Methylocystis parvus |
| Vnf | Methylomusa anaerophila |
| Vnf | Paenibacillus durus |
| Vnf | Phaeospirillum fulvum |
| Vnf | Rhodoblastus acidophilus |
| Vnf | Rhodopseudomonas palustris |
| Vnf | Ruminiclostridium hungatei |
| Vnf | Tolumonas lignilytica |
| Anf | Anaerocolumna jejuensis |
| Anf | Azotobacter vinelandii |
| Anf | Clostridium pasteurianum |
| Anf | Desulfosporosinus fructosivorans |
| Anf | Desulfovibrio desulfuricans |
| Anf | Dickeya paradisiaca |
| Anf | Dysgonomonas capnocytophagoides |
| Anf | Geobacter thiogenes |
| Anf | Methanosarcina acetivorans C2A |
| Anf | Pectinatus cerevisiiphilus |
| Anf | Phaeospirillum fulvum |
| Anf | Rahnella sp. AA |
| Anf | Rhodobacter capsulatus |
| Anf | Rhodoblastus acidophilus |
| Anf | Rhodopseudomonas palustris |
| Anf | Sporomusa termitida |
| Anf | Thiorhodococcus drewsii |
| BchChl | Acaryochloris sp. RCC1774 |
| BchChl | Acetobacteraceae bacterium DB1506 |
| BchChl | Acetobacteraceae bacterium KEBCLARHB70R |
| BchChl | Acidibrevibacterium fodinaquatile |
| BchChl | Acidiphilium multivorum |
| BchChl | Acidocella sp. 20-57-95 |
| BchChl | Acuticoccus kandeliae |
| BchChl | Aestuariivita boseongensis |
| BchChl | Afifella marina |
| BchChl | Agrobacterium albertimagni |
| BchChl | Ahrensia sp. R2A130 |
| BchChl | Albimonas pacifica |
| BchChl | Aliterella atlantica |
| BchChl | Alkalinema sp. CACIAM 70d |
| BchChl | Allochromatium vinosum |
| BchChl | alpha proteobacterium AAP38 |
| BchChl | Alphaproteobacteria bacterium |
| BchChl | Alphaproteobacteria bacterium HGW-Alphaproteobacteria-1 |
| BchChl | Alphaproteobacteria bacterium HGW-Alphaproteobacteria-14 |
| BchChl | Alphaproteobacteria bacterium PA4 |
| BchChl | Alphaproteobacteria bacterium WS11 |
| BchChl | Altererythrobacter ishigakiensis |
| BchChl | Alteromonadaceae bacterium |
| BchChl | Anabaena cylindrica |
| BchChl | Aphanocapsa feldmannii 288cV |
| BchChl | Aphanothece hegewaldii |
| BchChl | Aquabacterium sp. W35 |
| BchChl | Aquidulcibacter paucihalophilus |
| BchChl | Aquincola tertiaricarbonis |
| BchChl | Arthrospira platensis |
| BchChl | Ascidiaceihabitans donghaensis |
| BchChl | Aurantimonas sp. 22II-16-19i |
| BchChl | Azospirillum |
| BchChl | bacterium RmlP026 |
| BchChl | Beijerinckiaceae bacterium RH AL1 |
| BchChl | Belnapia moabensis |
| BchChl | beta proteobacterium AAP51 |
| BchChl | beta proteobacterium AAP99 |
| BchChl | Betaproteobacteria bacterium HGW-Betaproteobacteria-3 |
| BchChl | Betaproteobacteria bacterium TMED41 |
| BchChl | Betaproteobacteria bacterium TMED82 |
| BchChl | Betaproteobacteria bacterium TMED156 |
| BchChl | Betaproteobacteria bacterium UKL13-2 |
| BchChl | Blastochloris sp. GI |
| BchChl | Blastomonas natatoria |
| BchChl | Bosea sp. AAP35 |
| BchChl | Bradyrhizobiaceae bacterium |
| BchChl | Bradyrhizobium guangzhouense |
| BchChl | Brevundimonas bacteroides |
| BchChl | Burkholderiales bacterium |
| BchChl | Burkholderiales bacterium 28-67-8 |
| BchChl | Burkholderiales bacterium C2 |
| BchChl | Burkholderiales bacterium PBB1 |
| BchChl | Burkholderiales bacterium PBB2 |
| BchChl | Burkholderiales bacterium RIFCSPHIGHO2_12_FULL_69_20 |
| BchChl | Caenispirillum salinarum |
| BchChl | Calothrix brevissima |
| BchChl | Candidatus Atelocyanobacterium thalassa |
| BchChl | Candidatus Chloroploca sp. Khr17 |
| BchChl | Candidatus Oscillochloris fontis |
| BchChl | Candidatus Phycosocius bacilliformis |
| BchChl | Candidatus Synechococcus spongiarum |
| BchChl | Candidatus Thermochlorobacter aerophilum |
| BchChl | Candidatus Thiodictyon syntrophicum |
| BchChl | Candidatus Viridilinea mediisalina |
| BchChl | Caulobacteraceae bacterium PMMR1 |
| BchChl | Cereibacter changlensis |
| BchChl | Chamaesiphon minutus |
| BchChl | Chloracidobacterium thermophilum |
| BchChl | Chlorobaculum limnaeum |
| BchChl | Chlorobium limicola |
| BchChl | Chloroflexales bacterium ZM16-3 |
| BchChl | Chloroflexus aggregans |
| BchChl | Chlorogloea sp. CCALA 695 |
| BchChl | Chlorogloeopsis fritschii |
| BchChl | Chloroherpeton thalassium |
| BchChl | Chondrocystis sp. NIES-4102 |
| BchChl | Chromatiaceae bacterium |
| BchChl | Chromatium okenii |
| BchChl | Chromatocurvus halotolerans |
| BchChl | Chroococcidiopsis sp. TS-821 |
| BchChl | Chrysosporum ovalisporum |
| BchChl | Citromicrobium sp. WPS32 |
| BchChl | Cognatiyoonia koreensis |
| BchChl | Coleofasciculus chthonoplastes |
| BchChl | Comamonadaceae bacterium |
| BchChl | Comamonadaceae bacterium SYSU G00088 |
| BchChl | Comamonadaceae bacterium YIM 73032 |
| BchChl | Congregibacter litoralis |
| BchChl | Crinalium epipsammum |
| BchChl | Cuspidothrix issatschenkoi |
| BchChl | Cyanobacteria bacterium J007 |
| BchChl | Cyanobacteria bacterium J083 |
| BchChl | Cyanobacteria bacterium UBA12227 |
| BchChl | Cyanobacterium aponinum |
| BchChl | cyanobacterium endosymbiont of Rhopalodia gibberula |
| BchChl | cyanobacterium PCC 7702 |
| BchChl | Cyanobium gracile |
| BchChl | Cyanothece sp. BG0011 |
| BchChl | Cylindrospermum sp. NIES-4074 |
| BchChl | Dactylococcopsis salina |
| BchChl | Dankookia rubra |
| BchChl | Desertifilum sp. IPPAS B-1220 |
| BchChl | Dichotomicrobium thermohalophilum |
| BchChl | Dinoroseobacter shibae |
| BchChl | Dolichospermum planctonicum |
| BchChl | Dongia mobilis |
| BchChl | Ectothiorhodosinus mongolicus |
| BchChl | Ectothiorhodospira magna |
| BchChl | Elioraea sp. PF-30 |
| BchChl | Erythrobacter litoralis |
| BchChl | Euhalothece natronophila |
| BchChl | filamentous cyanobacterium CCP3 |
| BchChl | filamentous cyanobacterium ESFC-1 |
| BchChl | Filomicrobium sp. |
| BchChl | Fischerella sp. NIES-4106 |
| BchChl | Flavimaricola marinus |
| BchChl | Fortiea contorta |
| BchChl | Fulvimarina manganoxydans |
| BchChl | gamma proteobacterium HIMB55 |
| BchChl | gamma proteobacterium NOR5-3 |
| BchChl | Geitlerinema sp. PCC 7407 |
| BchChl | Geminicoccaceae bacterium |
| BchChl | Geminocystis herdmanii |
| BchChl | Gemmatimonadales bacterium |
| BchChl | Gemmatimonas phototrophica |
| BchChl | Gemmobacter sp. CC-PW-75 |
| BchChl | Gloeobacter kilaueensis |
| BchChl | Gloeocapsa sp. PCC 7428 |
| BchChl | Gloeocapsopsis sp. AAB1 = 1H9 |
| BchChl | Gloeomargarita lithophora |
| BchChl | Gloeothece citriformis |
| BchChl | Halieaceae bacterium |
| BchChl | Halomicronema hongdechloris |
| BchChl | Halorhodospira halochloris |
| BchChl | Halothece sp. PCC 7418 |
| BchChl | Hasllibacter halocynthiae |
| BchChl | Hassallia byssoidea VB512170 |
| BchChl | Heliobacillus mobilis |
| BchChl | Heliobacterium modesticaldum |
| BchChl | Heliophilum fasciatum |
| BchChl | Histidinibacterium lentulum |
| BchChl | Hoeflea olei |
| BchChl | Hormoscilla sp. GUM007 |
| BchChl | Hwanghaeicola aestuarii |
| BchChl | Hydrococcus rivularis |
| BchChl | Hydrogenophaga flava |
| BchChl | Hyella patelloides |
| BchChl | Hyphomicrobium sp. |
| BchChl | Hyphomicrobium sp. 12-62-95 |
| BchChl | Hyphomicrobium zavarzinii |
| BchChl | Hyphomonadaceae bacterium UKL13-1 |
| BchChl | Ideonella sakaiensis |
| BchChl | Imhoffiella purpurea |
| BchChl | Jannaschia aquimarina |
| BchChl | Kamptonema |
| BchChl | Kandeliimicrobium roseum |
| BchChl | Labrenzia alexandrii |
| BchChl | Leptolyngbya boryana |
| BchChl | [Leptolyngbya] sp. JSC-1 |
| BchChl | Leptothrix mobilis |
| BchChl | Limimaricola pyoseonensis |
| BchChl | Limnohabitans sp. 2KL-17 |
| BchChl | Limnoraphis robusta |
| BchChl | Limnothrix rosea |
| BchChl | Litoreibacter ponti |
| BchChl | Loktanella atrilutea |
| BchChl | Luminiphilus syltensis |
| BchChl | Lutimaribacter saemankumensis |
| BchChl | Lyngbya sp. PCC 8106 |
| BchChl | Maliponia aquimaris |
| BchChl | Maribius sp. MOLA 401 |
| BchChl | marine gamma proteobacterium HTCC2080 |
| BchChl | Marinibacterium profundimaris |
| BchChl | Marinovum sp. |
| BchChl | Maritimibacter sp. |
| BchChl | Marivita geojedonensis |
| BchChl | Mastigocladopsis repens |
| BchChl | Mastigocoleus testarum |
| BchChl | Merismopedia glauca |
| BchChl | Mesorhizobium loti |
| BchChl | Methylibium sp. NZG |
| BchChl | Methylobacterium brachiatum |
| BchChl | Methylocapsa palsarum |
| BchChl | Methylocella silvestris |
| BchChl | Methylocystis rosea |
| BchChl | Methylorubrum extorquens |
| BchChl | Methyloversatilis sp. RAC08 |
| BchChl | Microcoleus sp. PCC 7113 |
| BchChl | Microcystis aeruginosa |
| BchChl | Mongoliimonas terrestris |
| BchChl | Moorea producens |
| BchChl | Myxococcales bacterium |
| BchChl | Myxosarcina sp. GI1 |
| BchChl | Neosynechococcus sphagnicola |
| BchChl | Nereida ignava |
| BchChl | Nevskia ramosa |
| BchChl | Niveispirillum lacus |
| BchChl | Nodosilinea nodulosa |
| BchChl | Nodularia sp. NIES-3585 |
| BchChl | Nostoc calcicola |
| BchChl | Nostocales cyanobacterium |
| BchChl | Nostocales cyanobacterium HT-58-2 |
| BchChl | Novosphingobium acidiphilum |
| BchChl | Oceanibaculum indicum |
| BchChl | Oceanicola sp. HL-35 |
| BchChl | Oceanobacter sp. |
| BchChl | Okeania hirsuta |
| BchChl | Oscillatoria acuminata |
| BchChl | Oscillatoria nigro-viridis |
| BchChl | Oscillatoriales cyanobacterium CG2_30_44_21 |
| BchChl | Oscillatoriales cyanobacterium JSC-12 |
| BchChl | Oscillatoriales cyanobacterium USR001 |
| BchChl | Oscillochloris trichoides |
| BchChl | Palleronia abyssalis |
| BchChl | Paracraurococcus ruber |
| BchChl | Pararhizobium sp. BGMRC6574 |
| BchChl | Pararhodospirillum oryzae |
| BchChl | Pelagicola sp. LXJ1103 |
| BchChl | Pelodictyon luteolum |
| BchChl | Pelomonas puraquae |
| BchChl | Phaeobacter sp. 22II1-1F12B |
| BchChl | Phaeospirillum fulvum |
| BchChl | Phormidesmis priestleyi |
| BchChl | Phormidium ambiguum |
| BchChl | Phreatobacter oligotrophus |
| BchChl | Phyllobacteriaceae bacterium |
| BchChl | Phyllobacteriaceae bacterium StC1 |
| BchChl | Phyllobacteriaceae bacterium Z3-1 |
| BchChl | Piscinibacter caeni |
| BchChl | Planktotalea frisia |
| BchChl | Planktothricoides sp. SR001 |
| BchChl | Planktothrix sp. PCC 11201 |
| BchChl | Polymorphobacter sp. DJ1R-1 |
| BchChl | Polynucleobacter acidiphobus |
| BchChl | Ponticoccus marisrubri |
| BchChl | Pontivivens insulae |
| BchChl | Porphyrobacter colymbi |
| BchChl | Primorskyibacter sp. SS33 |
| BchChl | Prochlorococcus marinus |
| BchChl | Prochlorothrix hollandica |
| BchChl | Propionibacteriaceae bacterium |
| BchChl | Prosthecochloris marina |
| BchChl | Prosthecomicrobium hirschii |
| BchChl | Proteobacteria bacterium SG_bin5 |
| BchChl | Proteobacteria bacterium SG_bin6 |
| BchChl | Proteobacteria bacterium ST_bin13 |
| BchChl | Proteobacteria bacterium ST_bin14 |
| BchChl | Pseudacidovorax sp. RU35E |
| BchChl | Pseudaestuariivita atlantica |
| BchChl | Pseudanabaena biceps |
| BchChl | Pseudohaliea rubra |
| BchChl | Pseudomonas sp. SLBN-2 |
| BchChl | Pseudooctadecabacter jejudonensis |
| BchChl | Pseudorhodobacter sp. MZDSW-24AT |
| BchChl | Pseudorhodoferax sp. Leaf267 |
| BchChl | Puniceibacterium antarcticum |
| BchChl | Rhizobacter sp. Root404 |
| BchChl | Rhizobiales bacterium |
| BchChl | Rhizobiales bacterium 65-79 |
| BchChl | Rhizobiales bacterium CCH10-E5 |
| BchChl | Rhizobiales bacterium KCTC 52945 |
| BchChl | Rhizobium ipomoeae |
| BchChl | Rhodobacter capsulatus |
| BchChl | Rhodobacteraceae bacterium |
| BchChl | Rhodobacteraceae bacterium CCMM004 |
| BchChl | Rhodobacteraceae bacterium CG17_big_fil_post_rev_8_21_14_2_50_63_15 |
| BchChl | Rhodobacteraceae bacterium EhC02 |
| BchChl | Rhodobacteraceae bacterium HLUCCA08 |
| BchChl | Rhodobacteraceae bacterium HLUCCA09 |
| BchChl | Rhodobacteraceae bacterium HLUCCO18 |
| BchChl | Rhodobacteraceae bacterium JBTF-M29 |
| BchChl | Rhodobacteraceae bacterium LMIT002 |
| BchChl | Rhodobacteraceae bacterium MA-7-27 |
| BchChl | Rhodobacteraceae bacterium O448 |
| BchChl | Rhodobacteraceae bacterium PARR1 |
| BchChl | Rhodobacteraceae bacterium SB2 |
| BchChl | Rhodobacteraceae bacterium SM1902 |
| BchChl | Rhodobacteraceae bacterium TG-679 |
| BchChl | Rhodobacteraceae bacterium THAF1 |
| BchChl | Rhodobacteraceae bacterium WDS1C4 |
| BchChl | Rhodobacteraceae bacterium WDS4C29 |
| BchChl | Rhodobacterales bacterium |
| BchChl | Rhodobium orientis |
| BchChl | Rhodoblastus acidophilus |
| BchChl | Rhodoferax antarcticus |
| BchChl | Rhodoligotrophos appendicifer |
| BchChl | Rhodomicrobium sp. JA980 |
| BchChl | Rhodopila globiformis |
| BchChl | Rhodoplanes piscinae |
| BchChl | Rhodopseudomonas palustris |
| BchChl | Rhodosalinus sediminis |
| BchChl | Rhodospira trueperi |
| BchChl | Rhodospirillaceae bacterium |
| BchChl | Rhodospirillaceae bacterium HHTR118 |
| BchChl | Rhodospirillales bacterium |
| BchChl | Rhodospirillales bacterium 20-60-12 |
| BchChl | Rhodospirillales bacterium 20-64-7 |
| BchChl | Rhodospirillum centenum |
| BchChl | Rhodothalassium salexigens |
| BchChl | Rhodovibrio salinarum |
| BchChl | Rhodovulum adriaticum |
| BchChl | Richelia intracellularis |
| BchChl | Rippkaea orientalis |
| BchChl | Rivibacter subsaxonicus |
| BchChl | Rivularia sp. PCC 7116 |
| BchChl | Roseateles depolymerans |
| BchChl | Roseiarcus fermentans |
| BchChl | Roseibaca calidilacus |
| BchChl | Roseibacterium elongatum |
| BchChl | Roseibium hamelinense |
| BchChl | Roseicitreum antarcticum |
| BchChl | Roseicyclus mahoneyensis |
| BchChl | Roseiflexus castenholzii |
| BchChl | Roseinatronobacter monicus |
| BchChl | Roseisalinus antarcticus |
| BchChl | Roseitalea porphyridii |
| BchChl | Roseivivax halodurans |
| BchChl | Roseobacter denitrificans |
| BchChl | Roseofilum reptotaenium AO1-A |
| BchChl | Roseomonas nepalensis |
| BchChl | Roseospirillum parvum |
| BchChl | Roseovarius aestuariivivens |
| BchChl | Rubidibacter lacunae |
| BchChl | Rubrimonas cliftonensis |
| BchChl | Rubritepida flocculans |
| BchChl | Rubrivivax benzoatilyticus |
| BchChl | Ruegeria sp. PBVC088 |
| BchChl | Salinarimonas rosea |
| BchChl | Salinisphaera sp. Q1T1-3 |
| BchChl | Salipiger mucosus |
| BchChl | Sandarakinorhabdus limnophila |
| BchChl | Scytonema millei |
| BchChl | Shimia sp. WX04 |
| BchChl | Skermanella aerolata |
| BchChl | Snowella sp. |
| BchChl | Sphaerospermopsis |
| BchChl | Sphingobium sp. TLA-22 |
| BchChl | Sphingomonas astaxanthinifaciens |
| BchChl | Spirulina major |
| BchChl | Stanieria sp. NIES-3757 |
| BchChl | Stappia sp. ES.058 |
| BchChl | Streptomyces purpurogeneiscleroticus |
| BchChl | Sulfitobacter guttiformis |
| BchChl | Synechococcus elongatus |
| BchChl | Synechocystis sp. PCC 7509 |
| BchChl | Tabrizicola aquatica |
| BchChl | Tardiphaga sp. vice154 |
| BchChl | Tateyamaria omphalii |
| BchChl | Telmatospirillum siberiense |
| BchChl | Tepidamorphus gemmatus |
| BchChl | Thalassobacter stenotrophicus |
| BchChl | Thalassobaculum litoreum |
| BchChl | Thalassobium sp. R2A62 |
| BchChl | Thalassococcus profundi |
| BchChl | Thermosynechococcus elongatus |
| BchChl | Thiobaca trueperi |
| BchChl | Thiocapsa marina |
| BchChl | Thiocystis violascens |
| BchChl | Thioflavicoccus mobilis |
| BchChl | Thiohalocapsa sp. ML1 |
| BchChl | Thiorhodococcus drewsii |
| BchChl | Thiorhodospira sibirica |
| BchChl | Thiorhodovibrio sp. 970 |
| BchChl | Tolypothrix campylonemoides |
| BchChl | Tranquillimonas alkanivorans |
| BchChl | Trichodesmium erythraeum |
| BchChl | Trichormus azollae |
| BchChl | Tropicibacter sp. LMIT003 |
| BchChl | Tropicimonas sp. IMCC34043 |
| BchChl | Tychonema bourrellyi |
| BchChl | unclassified Betaproteobacteria (miscellaneous) |
| BchChl | unclassified Rhizobium |
| BchChl | unclassified Sphingomonas |
| BchChl | uncultured marine proteobacterium |
| BchChl | uncultured marine type-A Synechococcus 4O4 |
| BchChl | uncultured marine type-A Synechococcus 5B2 |
| BchChl | uncultured marine type-A Synechococcus GOM 3O12 |
| BchChl | uncultured marine type-A Synechococcus GOM 5D20 |
| BchChl | uncultured proteobacterium DelRiverFos13D03 |
| BchChl | unicellular cyanobacterium SU2 |
| BchChl | Variovorax sp. KK3 |
| BchChl | Vulcanococcus limneticus |
| BchChl | Xenococcus sp. PCC 7305 |
| BchChl | Yoonia litorea |

**Supplementary File 1c. Strains and plasmids used in this study**.**

| **Name** | **Type** | **Description** | **Source** |
| --- | --- | --- | --- |
| DJ | Strain | Wild-type (WT); Nif+ | Dennis Dean, Virginia Tech |
| DJ2102 | Strain | Strep-tagged WT NifD; Nif+ | Dennis Dean, Virginia Tech |
| DJ2278 | Strain | Δ*nifD*::KanR; Nif- | Dennis Dean, Virginia Tech |
| DJ884 | Strain | *nifD*R187I mutant; Nif+(slow); overexpresses NifH | Dennis Dean, Virginia Tech |
| AK022* | Strain | Δ*nifHDK*::KanR; Nif- | This study |
| Anc1A (“AK013")* | Strain | Δ*nifD*::*nifD*^Anc1A^; Nif+ | This study |
| Anc1B (“AK023”)* | Strain | Δ*nifHDK*::*nifHDK*^Anc1B^; Nif+ | This study |
| Anc2 (“AK014”)* | Strain | Δ*nifD*::*nifD*^Anc2^; Nif+ |  |
| pAG25 | Plasmid | KanR cassette (APH(3’)-I gene) + 400-bp *nifHDK* flanking homology regions, synthesized into XbaI/KpnI sites in pUC19; used to construct strain AK022 from DJ | This study |
| pAG13 | Plasmid | *nifD*^Anc1A^ + 400-bp *nifD* flanking homology regions, synthesized into XbaI/KpnI sites in pUC19; used to construct strain Anc1A from AK022 | This study |
| pAG19 | Plasmid | *nifHDK*^Anc1B^ + 400-bp *nifHDK* flanking homology regions, synthesized into XbaI/KpnI sites in pUC19; used to construct strain Anc1B from AK022 | This study |
| pAG14 | Plasmid | *nifD*^Anc2^ + 400-bp *nifD* flanking homology regions, synthesized into XbaI/KpnI sites in pUC19; used to construct strain Anc2 from AK022 | This study |

*Kaçar Lab strain designations indicated for reference

**Physical materials including bacterial strains and plasmids are available to the scientific community upon request.

**Supplementary File 1d. Primers used in this study.**

| **Primer** | **Sequence (5’ to 3’)** | **Description** |
| --- | --- | --- |
| 306_nifH_F | GCCGAACGTTCAAGTGGAAA | Forward primer, binds non-coding sequence upstream of *nifH; f*or PCR amplification of *nifHDK* and *nifH* sequencing |
| 307_nifH_R | AGAGCCAATCTGCCCTGTC | Reverse primer, binds non-coding sequence downstream of *nifH*; for *nifH* sequencing |
| 308_nifD_F | CACCCGTTACCCGCATATGA | Forward primer, binds non-coding sequence upstream of *nifD*; for *nifD* sequencing |
| 309_nifD_R | ACTCATCTGTGAACGGCGTT | Reverse primer, binds non-coding sequence downstream of *nifD*; for *nifD* sequencing |
| 310_nifK_F | GCTAACGCCGTTCACAGATG | Forward primer, binds non-coding sequence upstream of *nifK*; for *nifK* sequencing |
| 311_nifK_R | TCAGTTGGCCTTCGTCGTTG | Reverse primer, binds non-coding sequence downstream of *nifK*; for PCR amplification of *nifHDK* and *nifK* sequencing |
